# Complete Genome Assemblies for Two Single-Chromosome *Vibrio cholerae* Isolates, Strains 1154-74 (Serogroup O49) and 10432-62 (Serogroup O27)

**DOI:** 10.1128/genomeA.00462-15

**Published:** 2015-05-14

**Authors:** S. L. Johnson, A. Khiani, K. A. Bishop-Lilly, C. Chapman, M. Patel, K. Verratti, H. Teshima, A. C. Munk, D. C. Bruce, C. S. Han, G. Xie, K. W. Davenport, P. Chain, S. Sozhamannan

**Affiliations:** a Los Alamos National Laboratory (LANL), Los Alamos, New Mexico, USA; b Naval Medical Research Center (NMRC)-Frederick, Fort Detrick, Maryland, USA; c Henry M. Jackson Foundation, Bethesda, Maryland, USA; d Goldbelt Raven, LLC, Frederick, Maryland, USA

## Abstract

Here, we report the completed genome sequences for two non-O1/non-O139 *Vibrio cholerae* isolates. Each isolate has only a single chromosome, as opposed to the normal paradigm of two chromosomes found in all other *V. cholerae* isolates.

## GENOME ANNOUNCEMENT

*Vibrio cholerae* is a comma-shaped Gram-negative bacterium best known as the causative agent of cholera. Cholera represents an estimated burden of 1.4 to 4.3 million cases and 28,000 to 142,000 deaths per year worldwide (1). Historically, cholera outbreaks have been linked to *V. cholerae* O1 serogroup strains or its derivatives of the O37 and O139 serogroups. A genomic study on the 2010 Haitian cholera outbreak strains highlighted the putative role of non O1/non-O139 *V. cholerae* in causing cholera (2). In a recent study, we examined the genomic diversity of a large collection of *V. cholerae* strains belonging to different non-O1/non-0139 serogroups using whole-genome mapping (3). In that study, we found pervasive genetic and genomic structural diversity, including indels, duplications, and fusions of the usual two chromosomes. Here, we report the complete genome assemblies of two single-chromosome *V. cholerae* isolates.

Each genome was drafted and assembled using four data types: Illumina short-read, Roche 454 standard and long-insert reads, and PacBio long reads. Short-read and long-insert paired 454 data were assembled together in Newbler, and consensus sequences were computationally shredded into 2-kbp overlapping shreds. The Illumina short-read data were assembled with Velvet (4), and consensus sequences were computationally shredded into 1.5-kb overlapping shreds. The PacBio long-read data were assembled using Hierarchical Genome Assembly Process (HGAP) (5). Consensus sequences from HGAP were computationally shredded into 10-kbp overlapping pieces. All shreds were integrated using Phrap. Possible misassemblies were corrected and repeat regions verified using in-house scripts and manual editing in Consed (6–8). All genomes were assembled to finished-quality completion (9), and each assembly was annotated using an Ergatis-based (10) workflow with minor manual curation.

The finding of a single chromosome was independently verified by whole-genome mapping and pulsed-field gel electrophoresis of intact chromosomes of these two strains (3). Due to the nonstandard topology of these genomes, we reviewed OpGen Argus-generated *in silico* optical maps for orthogonal confirmation of the single-chromosome nature of each genome.

The *V. cholerae* strain 1154-74 genome is 3.928 Mb (47.8% G+C content), and the *V. cholerae* strain 10432-62 genome is 4.077 Mb (47.7% G+C content). Annotation located 3,430 and 3,645 coding sequences, respectively, with similar gene profiles.

### Nucleotide sequence accession numbers. 

Annotated genome assemblies are publicly available in NCBI under accession numbers CP010811 (*V. cholerae* 1154-74) and CP010812 (*V. cholerae* 10432-62).
